# A deep intronic splice variant of the *COL4A5* gene in a Chinese family with X-linked Alport syndrome

**DOI:** 10.3389/fped.2022.1009188

**Published:** 2023-01-13

**Authors:** Pei Qian, Ying Bao, Hui-mei Huang, Lei Suo, Yan Han, Zhi-juan Li, Min Zhang

**Affiliations:** Department of Nephrology, Xi’an Children's Hospital, Xi'an, China

**Keywords:** Alport syndrome, COL4A5, splice site mutation, whole-genome sequencing, RT-PCR, minigene

## Abstract

**Background:**

X-linked Alport syndrome (XLAS) is caused by pathogenic variants in *COL4A5* and is characterized by progressive kidney disease, hearing loss, and ocular abnormalities.The aim of this study was to identify gene mutations in a Chinese family with XLAS, confirm a diagnosis, and provide an accurate genetic counseling.

**Methods:**

The proband was a 5-year-old male with microscopic hematuria and a family history of renal disease in 5 relatives.His relatives had microhematuria with or without proteinuria. His maternal uncle developed renal failure at the age of 35 years. He was evaluated by renal biopsy,whole-exome sequencing (WES) and whole-genome sequencing (WGS) for Alport syndrome. RT-PCR and cDNA Sanger sequencing were performed on RNA extracted from the skin of the proband. Then, a splicing reporter minigene assay was used to examine the effect of the variation on the splicing of the primary transcript in transfected cells.

**Results:**

Pathological examination of the kidney of the proband revealed diffuse thinning of the glomerular basement membrane, and immunofluorescence analysis indicated normal expression of the *α*5 chain in the basement membrane. No phenotype-associated candidate variant was detected in the proband *via* WES. A novel deep intronic *COL4A5* variant (c.385–716G > A), which is segregated with disease in this family, was identified using WGS. In-vitro minigene assay and in-vivo RT-PCR analysis demonstrated that the variant could produce both normal and abnormal transcripts. The abnormal transcripts showed that the variant activated a cryptic splice site, introducing a 147 bp pseudoexon into the mRNA sequence and consequently generating a premature termination codon (*p*.G129Afs*38) and leading to frameshifting and truncation of the *α*5 (collagen IV) protein.

**Conclusion:**

This is the first report of the novel c.385–716G > A splicing mutation in the COL4A5 gene, which illustrates the importance of performing WGS to find additional mutations in WES-negative patients with highly suspected forms of genetic diseases. The same results obtained from the in-vitro and in-vivo splicing experiments confirm the consistency between the minigene assay and RT-PCR analysis. In addition, this study highlights the importance of functional analysis in diagnosis and genetic counseling in AS.

## Introduction

Alport syndrome (AS) is a rare genetic disease of the glomerular basement membrane (GBM) caused by mutations in the *COL4A3*, *COL4A4*, and *COL4A5* genes, with a prevalence ranging from 1 in 5,000 to 1 in 53,000 individuals in different populations (1). Microhematuria is the most common symptom, and some patients will gradually develop proteinuria; most cases of AS will progress to end-stage renal disease (ESRD) by 30 years of age (2). The pathogenic variant of the COL4A5 gene (NM: 000495.5), which encodes *α*5 chain (collagen IV), causes X-linked AS that accounts for approximately 85% of the cases of AS (3). Some experts have recently asserted that patients with hematuria, thin basement membrane nephropathy (TBMN), and heterozygous mutations in *COL4A3* or *COL4A4* should be classified as cases of autosomal Alport syndrome (4). XLAS will not be the most frequent form of Alport syndrome if named in that manner. As in many heritable diseases, there is vast intra- and inter-familial variability and allelic heterogeneity in the phenotypic expression of XLAS, such as in disease severity, onset age, and progression (5). In male XLAS patients, the genotype-phenotype correlation is evident; whereas patients with the nonsense variants show the most severe, early-onset ESRD phenotype, those with a splice variant and missense variant show moderate and mild phenotypes, respectively (6). A previous study reported that heterozygous female patients usually have a milder and more variable clinical course compared with male patients with XLAS (7). However, a retrospective analysis of female Japanese patients with XLAS revealed that phenotypes in these patients may be severe and that 15% of patients reached ESRD by the age of 40 years (8). So, genetic testing is valuable for conformation of clinical diagnosis, providing prognostic advice and genetic counseling.

In recent years, Next-generation sequencing (NGS) has greatly improved the rate of diagnosis in XLAS. Despite these technological advances, at least one-fifth of XLAS patients still have no clearly defined molecular diagnosis (9), and this deficiency hampers a deeper understanding of the genotype–phenotype relationship in patients with XLAS. Standard NGS data analysis focuses on coding exons and splice sites and can miss the mutations in deep introns and promoters. However, Whole-genome sequencing (WGS) not only comprehensively covers exons but also detects deep intronic and intergenic variations, but interpretation of non-coding variants *via* WGS is challenging. It has been shown that splice variants account for approximately 15% of all cases of XLAS (6). It was found significant differences in renal survival that are based on the abnormal splicing patterns of the *COL4A5* primary transcript (6). However, the functional outcomes caused by many splice-region variants have not been investigated in AS patients. Further functional studies on the variations in *COL4A5* that may affect splicing are therefore necessary to identify the aberrant splicing patterns of the *COL4A5* primary transcript, define the relationship between the genotype and clinical phenotype, and predict the renal prognosis in AS. In this report, we identified a novel deep intronic variant of the *COL4A5* gene *via* WGS that could not have been detected by WES alone. Via a combination of these in-vivo, in-silico, and in-vitro analyses, the variant was found to impair the splicing process. After functional analysis, we concluded that this deep intronic variant in *COL4A5* is responsible for this family.

## Materials and methods

### Clinical data

**Figure 1:**
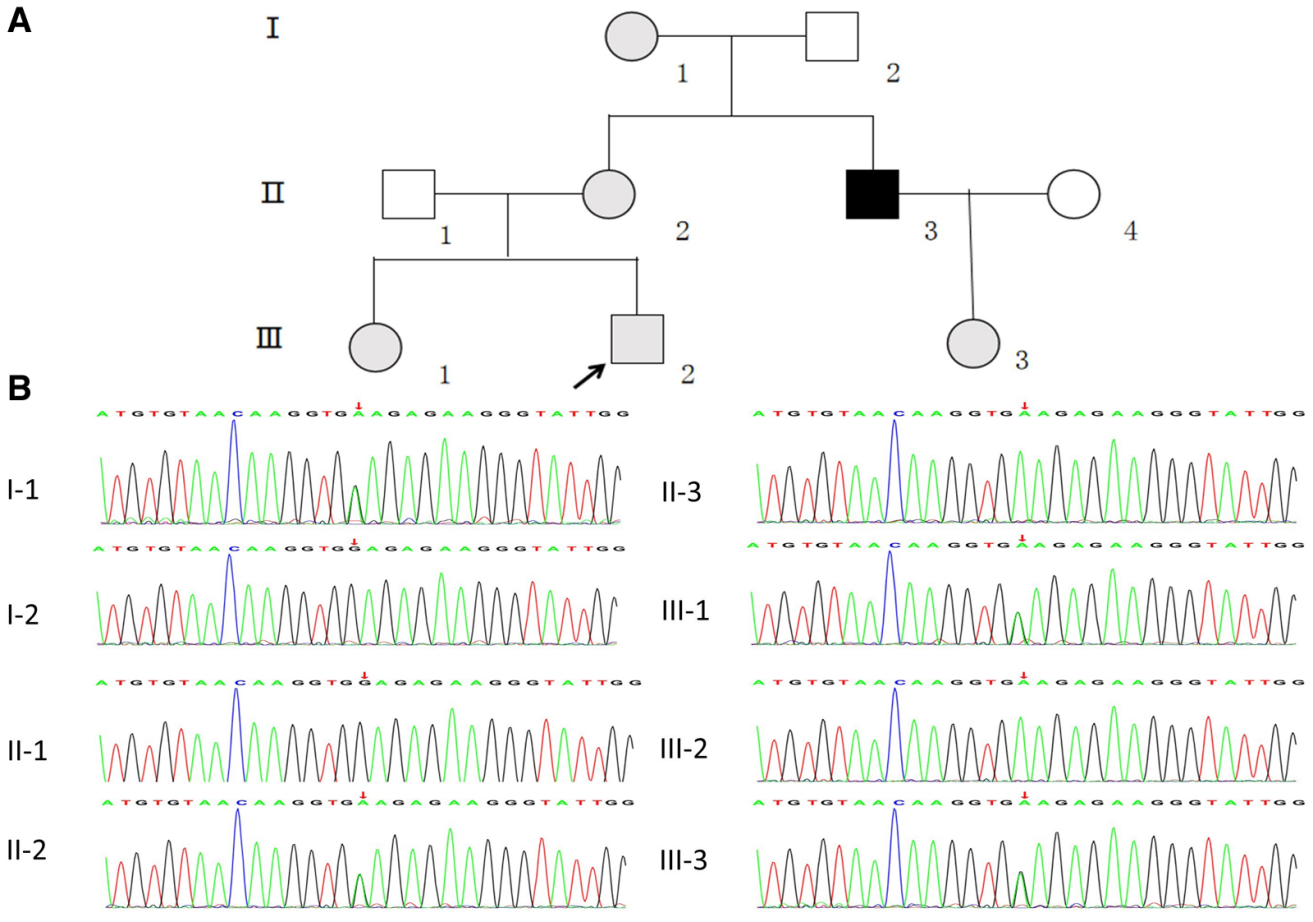
(**A**) pedigree analysis of the family. The gray, black, and white symbols represent the individuals with hematuria or proteinuria, renal failure, and healthy renal physiology, respectively. The proband is denoted by the black arrow. (**B**) Sequencing results showing the *COL4A5* mutations in the proband and his family members.

The study subjects included members of a Chinese family who is a 3-generation pedigree (Figure 1A and Table 1). The proband (III-2) was a 5-year-old boy., who was found to have microhematuria, but no proteinuria. No abnormalities were found in routine blood, serum chemical, or immunological tests. The results of pure-tone audiometry and eye examination were normal. Abdominal ultrasound examination, vascular color ultrasound, and electrocardiogram of the left renal vein showed no obvious abnormalities. His mother (II-2) presented with microhematuria and proteinuria with normal renal function, her quantity of proteinuria was 10∼15 mg/kg/24 h. His sister (III-1), older female cousin (III-3), and grandmother (I-1) all presented with microhematuria with normal renal function. His maternal uncle (II-3) presented with microhematuria and proteinuria and developed stage 5 chronic kidney disease by the age of 35 years, his quantity of proteinuria was 35∼40 mg/kg/24 h. Except for the proband's uncle, all the others had normal kidney function and blood pressure. In addition, the maternal uncle (II-3) and mother (II-2) of the proband had high-frequency sensorineural hearing impairment. Because the proband had long lasting microscopic hematuria and a family history of renal disease in 5 relatives, a kidney biopsy was performed on the proband due to the suspicion of AS.

**Table 1:** Clinical features of affected individuals in the family.

Pedigree	Sex	Ocular examination	Audiometry	Renal manifestations
I-1	Female	Normal	Normal	Microhematuria
II-2	Female	Normal	High-frequency sensorineural hearing impairment	Microhematuria, proteinuria
II-3	Male	Normal	High-frequency sensorineural hearing impairment	Microhematuria, proteinuria Progressed to CKD 5 by age 35
III-1	Female	Normal	Normal	Microhematuria
III-2	Male	Normal	Normal	Microhematuria: age 3 years Kidney biopsy: Thin GBM < 180 nm
III-3	Female	Normal	Normal	Microhematuria

CKD, chronic kidney disease; GBM, glomerular basement membrane.

### NGS and direct sanger sequencing

We collected 4 ml of peripheral blood samples from the proband and his family members for WES and WGS. Sanger sequencing was used to confirm the candidate variant. Genomic DNA samples were fragmented to approximately 200–300 bp by using ultrasound, and DNA libraries were prepared using the established Illumina paired-end protocol. The xGen Exome Research Panel v1.0 (Integrated DNA Technologies) was used as the whole-exon capture chip in the WES, which was performed in an Illumina NovaSeq 6,000 Series Sequencer (PE150). Whole-genome libraries were prepared according to the standardized on-machine sequencing operation procedures of the DNBSEQ-T7 platform. The raw sequencing data were aligned against the reference human genome (hg19) by using the BWA software. Repeated reads were excluded, and then statistical analysis was performed on the remaining reads. The GATK software was used to analyze SNPs and indels. According to the sequencing and mutation quality, the detected SNPs and indels were filtered and screened for high-quality and reliable mutations. For the detected high-quality variants, self-developed variant-annotation software was used to perform association annotation at major databases, such as OMIM, HGMD, ClinVar, and frequency databases, including dbSNP, 1,000 Genomes, ExAC, and ESP. Via protein structure prediction software, such as Provean, SIFT, Polyphen2-HVAR, Polyphen2-HDIV, M-Cap, Revel, Mutationtster, and MaxEntScan cleavage site prediction software, the hazards were analyzed, and the possible harmful effects on the protein structure were screened out variation. Variant loci were graded for pathogenicity according to the American College of Medical Genetics and Genomics (ACMG) guidelines (10). Copy number variation (CNV) detection was performed using self-developed CNV software. The genes included in the CNV interval were analyzed; related data, such as those from Decipher, ClinVar, OMIM, DGV, and ClinGen, were associated; and the reported disease genes were annotated. According to the annotation information and frequency database, the hazard level of CNV was comprehensively assessed. Sanger sequencing was performed on the samples of all available family members based on the results from NGS.

### In silico splicing assay

We used publicly available in silico analysis tools to predict the effects of the c.385–716G > A variant on the splicing of *COL4A5* exon 6. These tools were Human Splicing Finder [http://www.umd.be/HSF3/HSF.html], BDGP: Splice Site Prediction by Neural Network [http://www.fruitfly.org/seq_tools/splice.html], and NetGene2 Server [http://www.cbs.dtu.dk/services/NetGene2/].

### Hybrid minigene assay

We used a minigene plasmid based on the pMini-CopGFP vector (Beijing Hitrobio Biotechnology Co., Ltd., Beijing, China). Briefly, we cloned DNA fragments into pMini-CopGFP by using GenScript (Piscataway, NJ), and the BamHI and XhoI restriction sites around the target variations in the *COL4A5* gene (both wild-type [WT] and mutant-type [MT] with exons and introns) according to the manufacturer's instructions (primers available upon request).The hybrid minigenes were confirmed by sequencing and then transfected into 293 T cells by using Lipofectamine™ 2,000 (Thermo Fisher Scientifc, Waltham, MA, United States). The cells were harvested 24–48 h later, and total RNA was extracted using the RNeasy Plus Mini Kit (QIAGEN, Hilden, Germany). Subsequently, 1.5 µg of the total RNA was used to generate cDNA by using Multiscribe RT Polymerase (AB systems) according to the manufacturer's instructions. pMini-CopGFP-specific primers (available upon request) were used to amplify cDNA to determine the splicing patterns. The PCR products were analyzed using electrophoresis on a 1.5% agarose gel, followed by direct sequencing. The amplicon included *COL4A5* exons 6–8 in the case of normal splicing normal.

### Reverse transcription (rt)-PCR analysis

We obtained a skin sample from the proband. Total RNA was extracted from the skin sample and a blood sample derived from a healthy control, and then subjected to RT-PCR for COL4A5 primary transcript. Total RNA was isolated using TRIzol Reagent (Invitrogen, Carlsbad, CA). Then, 1 *μ*g of total RNA was reverse-transcribed to generate cDNA by using PrimeScript RT Reagent Kit with gDNA Eraser (TaKaRa Biotechnology (Dalian) Co., Ltd., Dalian, China). Duplex PCR was performed (primers available upon request) to amplify a 985 bp fragment of the *COL4A5* cDNA encompassing exons 1–17. Then, the PCR products were directly sequenced.

## Results

### Histopathology of renal biopsy

**Figure 2:**
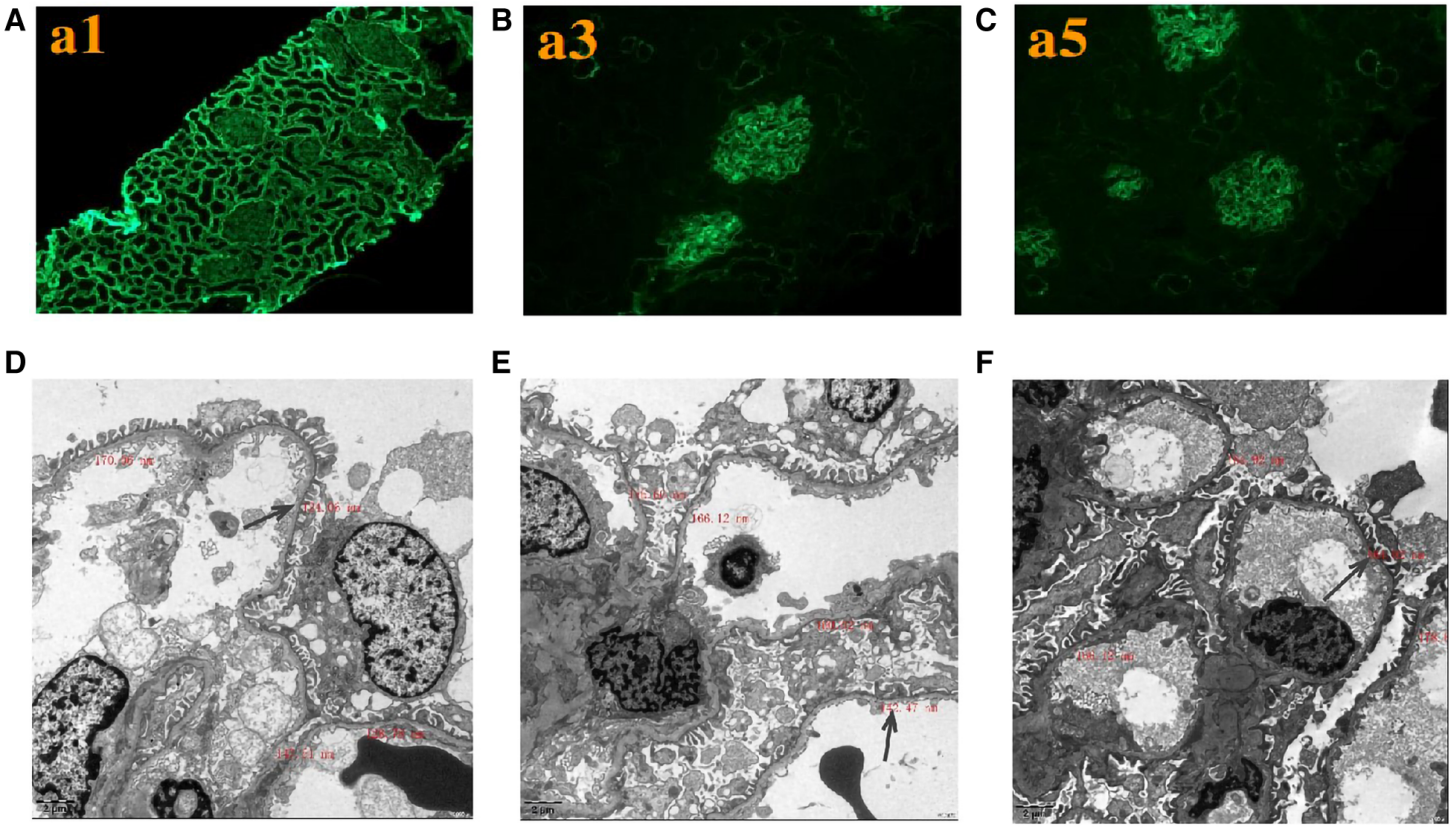
Renal expression patterns of *α*1, *α*3, and *α*5 and renal ultrastructure of the proband. Immunofluorescence staining of the kidney for (**A**) *α*1, (**B**) *α*3, and (**C**) *α*5. × 200. Electron microscopy images of his kidney show that the thickness of the basement membrane ranges around 124.06 nm (**D**), 142.47 nm (**E**), and 164.92 nm (**F**). EM × 50,000.

Renal histopathological features of proband are displayed in Figure 2. No glomerular immunoglobulin or complement component was observed *via* immunofluorescence staining. The expression patterns of the positive control (collagen IV *α*1 chain), collagen IV *α*3 chain and collagen IV *α*5 chain were normal. Light microscopy showed slight proliferation of mesangial cells and stroma, but no crescents were observed. The vacuolar degeneration of the capillary endothelial cells was observed *via* electron microscopy (EM). The parietal layer of the renal follicle was not significantly thickened, and the basement membrane was diffusively thinned to a thickness of < 180 nm. Comprehensive light microscopy, immunofluorescence analyses, and EM showed that the glomerular lesions were mild, and the morphology indicated thin basement-membrane nephropathy.

### Mutation analysis

WES was performed first, but no candidate variant related to the phenotype was found. Thus, WGS was performed next since it has higher coverage than WES. The WGS test results revealed a novel hemizygous variant in intron 6 of the proband (chrX: 107813927, Ghg19, NM_000495.5: c.385–716G > A). He was found to inherit this variant from his mother. Pedigree validation *via* Sanger sequencing revealed that all the affected female patients (I-1, II-2, III-1, III-2, and III-3) and the affected male patient (the maternal uncle of the proband, II-3) in this pedigree were heterozygous for this variant (c.385–716G > A), whereas the asymptomatic family members did not carry the variant (Figure 1B). Additionally, the phenotypes and genotypes of the proband and the 5 other patients in the family were co-isolated. The discovered variant (c.385–716G > A) is a deep intronic variant (> 100 bp from the splice site), absent from the control databases or from the Single Nucleotide Polymorphism Database (dbSNP) and ClinVar database. Additionally, it has not been reported to be associated with any disease.

### In silico splicing analysis

No significant effect of this variant on splicing was detected by Human Splicing Finder and NetGene. The BDGP online software suggested that the variant produced a new splice donor site located in c.385–716G > A.

### In vitro splicing analysis

**Figure 3:**
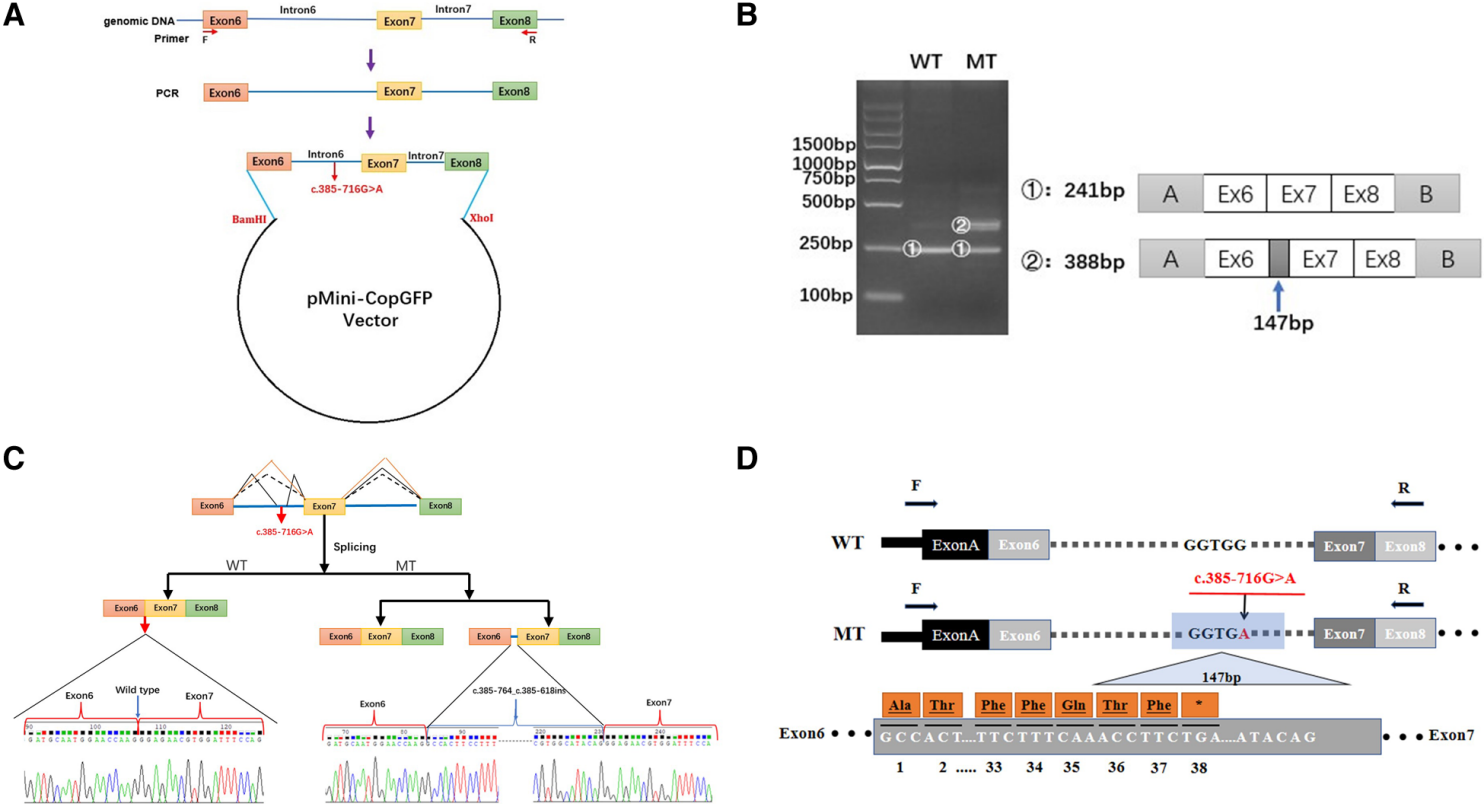
Minigene-assay transcript analysis. (**A**) *COL4A5* exons 6–8, spanning the intron variants, were cloned into the pMini-CopGFP vector. (**B**) Electrophoresis of the reverse transcription-polymerase chain reaction products of the minigene transcripts. The wild type (WT) exhibited a single, full-length band (241 bp), and the mutant type (MT) exhibited two bands (the WT band and 147 bp insert band). The drawing on the right shows the predicted WT and MT amplicon sizes. (**C**) Schematic diagram showing the variation and its consequences. The WT situation is indicated by the orange line. The variation produces two transcripts, produced *via* WT and MT splicing (indicated by black dashes and lines, respectively). The change of c.385-716G > A introduces a new splice site, resulting in a cryptic exon activation between exons 6 and 7 and creating a transcript with a 147 bp insertion. (**D**) The structure of the WT COL4A5 transcript fragment spanning exons 6–8 and that of the MT version, generated by the 385-716G > A variation. A 147 bp sequence around the variation is introduced to the mRNA to form a pseudo-exon between exons 6 and 7. A TGA termination codon appeared at 114 bp in the 147 bp fragment, resulting in translational termination of the frameshifted polypeptide after 37 amino acids are translated (modified and referenced from Verdura E (23)).

As the discovered variant is located in intron 6 of the *COL4A5* gene, we suspected that it might affect the splicing of the primary COL4A5 transcript. We amplified the COL4A5 DNA fragments spanning exons 6–8 and then cloned them into the minigene pMini-CopGFP vector (Figure 3A). Afterward, 293 T cells were transfected with the WT and MT splice-region variants for functional assessment (see Materials and Methods). Agarose gel electrophoresis (Figure 3B) showed that, after the intron sequence was spliced, the cells transfected with the WT vector yielded the expected 241-bp amplicon, containing COL4A5 exons 6–8, flanked by the exons of the vector. In contrast, the cells transfected with the MT vector produced two amplicons, one with the normal size of 241 bp, and the other with the size of 388 bp amplicon, with the additional 147 bp fragment added from exon 6 of COL4A5. The variant disrupts the splicing acceptor site of intron 6, preventing the removal of intron 6 from the primary transcript and thus resulting in the extra 147 bp. The change in the splicing results in the truncation of exon 6 from C.385–764 to C.385–618 (Figure 3C), and this new mRNA sequence contains a premature termination codon (*p*. G129Afs*38, Figure 3D).

### In vivo validation

**Figure 4:**
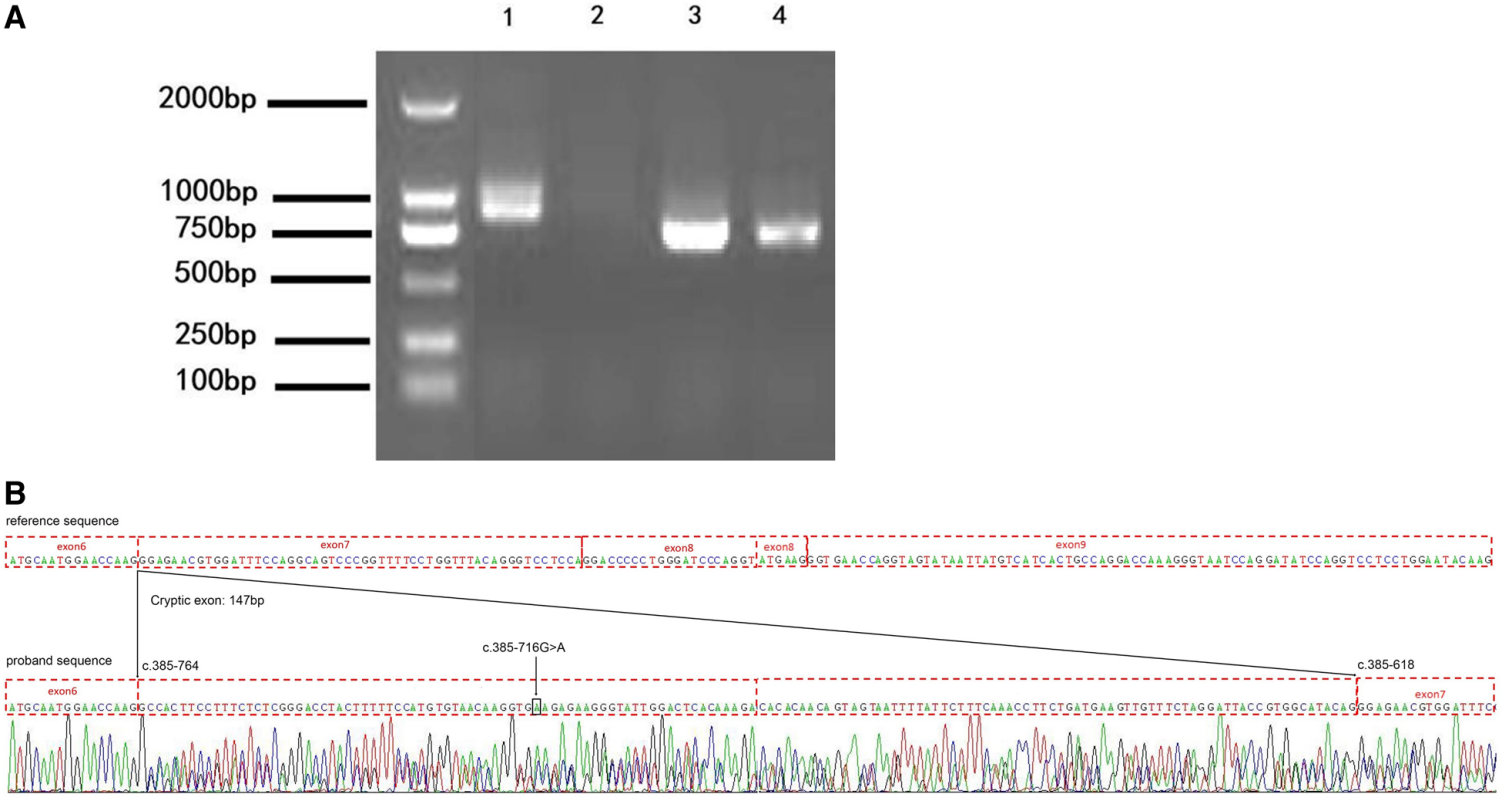
The variation (c.385-716G > A) perturbs the splicing of the *COL4A5* transcript. (**A**) Agarose gel electrophoresis results of the reverse transcription–polymerase chain reaction (RT-PCR) analysis of the patient (1 and 3, skin) and healthy control (2 and 4, blood) for the presence of the target (1 and 2) and reference (3 and 4) mRNAs (985 bp and 843 bp amplicon size, respectively). (**B**) Results of the Sanger sequencing of the RT-PCR products. c.385-716 G > A, black arrow and box position. The mutation introduces a 147 bp cryptic exon between exons 6 and 7 (c.385-764 and c.385-618 border exons 6 and 7, respectively).

To confirm the result from the minigene assay, we assessed the proband for the predicted splicing aberration. Gel electrophoresis (Figure 4A) showed the two predicted bands (WT and MT) from the skin sample of the proband, proving that the dermal cells of the proband produced the WT and MT COL4A5 mRNA variants. The doublet was purified from the gel and subjected to Sanger sequencing for confirmation. The results (Figure 4B) confirmed that the variant (c.385–716G > A) produces both the WT and MT transcripts. The MT transcript produces an abnormal mRNA that includes a 147 bp fragment from intron 6, a cryptic exon, and a premature termination codon (*p*. G129Afs*38). Because there is only a small size difference between the WT and MT transcripts, the electrophoretic bands form a doublet and are difficult to distinguish from each other. The results from our *in vitro* and *in vivo* splicing assays are consistent.

## Discussion

In this study, we reported a heterozygous deep intronic variant of the *COL4A5* gene (c.385–716G > A) through WGS, and this variant could not have been detected using WES alone. The variant initially was classified as “a variant of uncertain significance” according to the ACMG guidelines (PM2 + PP1-Moderate). However, based on its co-isolation with the family phenotype and our *in vitro* and *in vivo* results on its impact on the splicing of the *COL4A5* transcript, the mutation was finally classified as “pathogenic” (PM2 + PS3 + PP1-Strong). We concluded that this mutation is responsible for the disease of the proband, who was accordingly diagnosed with AS. All the affected family members carried this variant, and it was not detected in the father or maternal grandfather of the proband, who were unaffected. The maternal uncle of the proband developed ESRD at the age of 35 years, but the 80-year-old grandmother and 40-year-old mother of the proband had normal renal function when this research article was prepared. The male members of the family who carried the variant tended to have more severe phenotypes than the female members with the variant, indicating a typical X-linked inheritance pattern, and the severity of the disease varied among the pedigree individuals.

A growing number of diseases are attributed to abnormal splicing (11). Such variants reported by previous studies on splicing mutations are mostly located at evolutionarily conserved sites within exon–intron boundaries. However, few reports have focused on the effect of intron variation. In this study, we found that a deep intron variation in *COL4A5* can also lead to abnormal splicing. Some deep intronic variants of COL4A5 were found to exhibit pathogenicity by generating cryptic exons (12). These findings highlight the importance of identifying splice variants of *COL4A5*, rare functional intronic variants in particular. However, a consensus approach for detecting deep intronic variants has not been established (13). Although intron variations can be detected *via* WGS, it is difficult to distinguish intron variations that cause splicing errors from harmless polymorphisms. Currently, the best approach to identify pathogenic splicing mutations is to use a combination of these *in vivo*, in silico, and *in vitro* analyses. Although various in silico approaches have been developed to assess the effects of sequence variations, the most reliable approach to evaluating splicing pathogenicity is through *in vivo* analyses (14), which are based on analysis of the relevant transcripts directly in infected tissues. However, cDNA analysis of *COL4A5* using renal mRNA is not a common approach. It is often difficult to amplify *COL4A5* transcripts extracted from peripheral leukocytes, and analysis of transcripts *via* targeting genomic DNA variants is also unsatisfactory. Currently, when *in vivo* splicing studies are not available, the most effective method for functional analysis of intron variations is based on the hybrid minigene assay (13, 15). In this study, our in silico assessment predicted that the variation might affect the splicing, but the results from different algorithms were inconsistent. Subsequently, our in-vitro hybrid minigene assay and RT-PCR analysis of the skin cells from the patient confirmed that the variation perturbs the splicing; the variant vector could simultaneously express the WT and MT transcripts. The MT transcript introduced a 147 bp fragment from intron 6, a cryptic exon, and a premature termination codon (*p*. G129Afs * 38). This truncating mutation may result in a variable deposition of COL4A5-expressed proteins in the tissue. The degree of consistency between the minigene analysis and patient RNA analysis has not been fully established to date. Via transcript analysis of the patient skin, this study confirmed that the discovered variant perturbed the splicing, and both this in-vivo transcript analysis and in-vitro minigene assay yielded the same results.

The particular form of competitive splicing pattern that we detected in this study has been reported in other studies, with previous findings confirming the presence of both wild-type and abnormal transcripts leading to the truncated *COL4A5* variant in the kidney of a male patient with a splicing variant (16). In this study, none of the affected patients had abnormal eye or hearing abnormalities, except for the mother and maternal uncle of the proband. This observation may reflect the multiorgan-specific splicing pattern of *COL4A5* (16). In addition, the amount of the WT *COL4A5* transcript expressed may also vary among different cell types. The inner ear cells of some patients may not be able to produce *COL4A5* transcripts normally or may not splice the primary transcript correctly, resulting in the hearing loss observed in some members of this family. The clinical phenotypes of the female patients in this study were generally mild, these patients showed both normally and abnormally spliced transcripts, the frequency of normal *COL4A5* transcripts in the affected women may partly explain the differences in disease severity and phenotypic differences among the members of this family. In XLAS, men show more severe phenotypes than women due to the X-linked inheritance pattern. Men with XLAS can present with proteinuria and hematuria in early childhood and develop ESRD at a median age of 25–35 years (17). Regarding splice site mutations, Jais et al. have reported a 70% probability of ESRD development by the age of 30 (18). The mean onset age of ESRD in patients with splice-site variants is 28 (11). The median ages of ESRD in patients with a truncating splicing mutation and in those with a non-truncating splicing mutation have been reported to be 20 and 29 years old, respectively, indicating that non-truncating splicing variants cause more modest phenotypes than truncating ones (19). The fact that the renal outcomes of male XLAS patients with abnormal splicing differ depending on whether the mutations cause truncated or non-truncated proteins also suggests that for an accurate prognostic prediction, it is important to define transcript patterns when detecting splice-site mutations or abnormal splicing mutations. The mutation found in this study causes truncation of *α*5 (collagen IV). A previous study reported that the incomplete mutation of the splice site may produce both normal and abnormal kidney mRNA, leading to milder phenotypes (20). In this study, the maternal uncle of the proband developed renal failure by the age of 35 years, who exhibited a milder phenotype presumably because of producing a mixture of both WT and MT mRNAs. The normal transcript may have prevented the progression to the typically severe phenotype of XLAS. It has previously been reported that men with XLAS with positive a5 (IV) staining in the kidney often carry non-truncating mutations (mainly missense mutations) and show a milder clinical phenotype (21). Studies on patients with splicing site variation at the genomic level have found that positive *α*5 staining (collagen IV) in patient tissues indicates non-truncating transcriptional variation (19). In the present study, the proband showed positive a5 (IV collagen) staining in the kidney, but the transcript analysis revealed a truncating mutation, different from the non-truncating variation reported previously, which also calls into question the belief that renal immunohistochemical analysis for a5 (IV) predicts the genotype.

Although there is no specific treatment for AS, its management is aimed at delaying progression to renal failure using nephroprotective drugs (e.g., renin-angiotensin inhibitors). In this study, none of them received any treatment except the proband's uncle, who received peritoneal dialysis and hypertension treatment before diagnosis. It has been found that splicing regulation can induce a non-truncated transcription and produce a protein that partially compensates for the loss of the full-length protein, thereby improving the symptoms of the corresponding disease (22). Therapeutic approaches using splicing modulation that can alter truncating transcription to non-truncating transcription have been found effective in the treatment of XLAS (19). Understanding the differences between truncating non-sense mutations and non-truncating mutations is essential to assess renal prognosis, perform genetic counseling, and develop treatment strategies. Our study provides a foundation for the future gene therapy of this pathogenic variant.

## Conclusion

A novel pathogenic *COL4A5* deep intronic variant was identified in a Chinese family with AS through WGS, and its splicing effect was verified using the minigene assay and *in vivo* analysis. This study illustrates the importance of performing WGS to find additional mutations and confirm the consistency between the minigene assay and RT-PCR analysis. Differences in the proportions of the normal and abnormal transcripts in affected individuals carrying splice region variants may be responsible for the broad expressivity within this family, which provides insights into genotype-phenotype correlations.

## Data Availability

The datasets presented in this study can be found in online repositories. The names of the repository/repositories and accession number(s) can be found below: [INSERT REPOSITORY AND ACCESSION] Clinvar database (ClinVar Wizard Submission: SUB11982751).
